# The evolutionary ecology of fungal killer phenotypes

**DOI:** 10.1098/rspb.2023.1108

**Published:** 2023-08-30

**Authors:** Thomas J. Travers-Cook, Jukka Jokela, Claudia C. Buser

**Affiliations:** ^1^ Institute of Integrative Biology, ETH Zürich, Zürich, Switzerland; ^2^ Department of Aquatic Ecology, Eawag, Dübendorf, Switzerland

**Keywords:** allelopathy, coevolution, competition, symbiosis, toxin, virus

## Abstract

Ecological interactions influence evolutionary dynamics by selecting upon fitness variation within species. Antagonistic interactions often promote genetic and species diversity, despite the inherently suppressive effect they can have on the species experiencing them. A central aim of evolutionary ecology is to understand how diversity is maintained in systems experiencing antagonism. In this review, we address how certain single-celled and dimorphic fungi have evolved allelopathic killer phenotypes that engage in antagonistic interactions. We discuss the evolutionary pathways to the production of lethal toxins, the functions of killer phenotypes and the consequences of competition for toxin producers, their competitors and toxin-encoding endosymbionts. Killer phenotypes are powerful models because many appear to have evolved independently, enabling across-phylogeny comparisons of the origins, functions and consequences of allelopathic antagonism. Killer phenotypes can eliminate host competitors and influence evolutionary dynamics, yet the evolutionary ecology of killer phenotypes remains largely unknown. We discuss what is known and what remains to be ascertained about killer phenotype ecology and evolution, while bringing their model system properties to the reader's attention.

## 1. Introduction

Competition is often partitioned into subcategories of exploitation and interference competition, which respectively involve indirect resource-mediated and direct individual-level antagonism [1,2]. Interference strategies act as efficient alternatives to the generality of exploitation competition and are expected to evolve as trade-offs between the benefits of excluding competitors and the costs of attempts to do so [3–5]. Such benefits and costs are often context dependent [6], inviting phenotypic polymorphism and plasticity into antagonistic trait expression.

In its simplest form, interference competition should lead to monomorphic populations and single species communities. However, communities tend to be assemblages of phenotypically and genetically diverse individuals belonging to different species, even where antagonistic interactions are evident [7–9]. The explanation for the apparent persistence of diversity is that processes like interference competition select for competitor counter adaptations for mitigating against antagonism [10–12]. Logically, this process can be expected to trigger coevolutionary dynamics. The research challenge that remains is to evaluate whether coevolutionary dynamics are a common force maintaining polymorphism and diversity in populations and communities where interference competition is frequent [3,13].

Here, we propose fungal killer phenotypes as ideal models for studying the ecological and evolutionary consequences of interference competition. Fungi host diverse competitive strategies [14,15], but perhaps none are quite as striking as the antagonistic allelopathy defining killer phenotypes. In short, killer phenotypes are an expression of genes encoding proteinaceous toxins, that upon liberation from the producer can eliminate intraspecific and interspecific competitors [16]. Fungal killer phenotypes have the following advantages as model systems: (i) who kills whom is discernible [16–20], (ii) many killer phenotypes belong to well-studied model organisms [21–24], and (iii) the mechanistic bases of killers, killing and immunity are reasonably well understood [25–32]. Toxin production can be encoded on nuclear genes, or alternatively by viral symbioses involving interactions between multiple viral classes [33]. The multipartite viral endosymbiosis underlying some killer phenotypes provides an additional layer of interest, granting opportunity to study host-endosymbiont interactions and the eco-evolutionary feedbacks they encounter with host competition. In this paper, we review our current understanding of the ecology and evolution of fungal killer phenotypes. Our core goals are to demonstrate the relevance of this phenomenon to the study of general ecological and evolutionary processes, and detail unexplored territory in fungal killer phenotype research.

## 2. An overview of the fungal killer phenotype phenomenon

### (a) The discovery of fungal killer phenotypes

**Figure 1.**
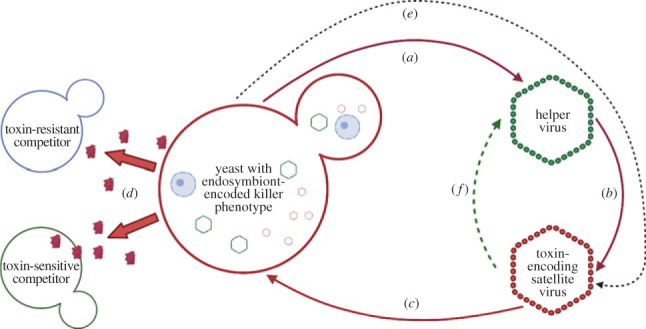
A basic schematic of the direct (solid lines) and indirect (dashed lines) uses of the parties to one another in a standard endosymbiont-based killer system. Endosymbiont-encoded killer systems involve three parties: the fungal host, the toxin-encoding satellite virus and the helper virus. (*a*) Helper viruses use host resources for their maintenance and replication. (*b*) Toxin-encoding satellite viruses rely on helper virus genes for their maintenance and replication in the fungal host cell. (*c*) Fungi process a preprotoxin that is encoded on the satellite virus genome. (*d*) Toxins are used against intraspecific and interspecific competitors, the benefits of which depend on the prevalence of resistance among competitors. (*e*) Toxin-encoding satellite viruses rely indirectly on their fungal hosts through their reliance on helper viruses. (*f*) Helper viruses rely indirectly on satellite viruses through the benefits satellites can provide to their shared host.

Killer phenotypes were first discovered in *Saccharomyces cerevisiae* by Bevan and Makower 60 years ago [16]*.* Strains of *S. cerevisiae* were classified as killers, sensitive or neutral (resistant), based on their survivorship in co-cultures. Killing was found to have an allelopathic basis, whereby specific abiotic conditions could activate proteinaceous toxin production [34]. Killer phenotypes were found to be encoded on non-mendelian genetic elements, later shown to be two distinct cytoplasmic double-stranded RNA (dsRNA) viruses [35,36]. Toxin-sensitive strains were found to lack the smaller virus (coined the M virus) and any observable killer phenotype, indicating its role in encoding toxin production and immunity [37,38]. The larger virus (coined the L-A virus) was found to autonomously replicate in the host, while the M virus could only be found in cells where the L-A virus was present [39]. Both viruses were found to be in possession of a L-A virus encoded major capsid protein (ScV-P1), revealing the dependence of the M virus on the L-A virus for its persistence in the host cell [40]. These findings together uncovered a multi-viral endosymbiosis underlying antagonistic allelopathy in fungi (figure 1).

### (b) The diversity of fungal killer phenotypes

**Figure 2.**
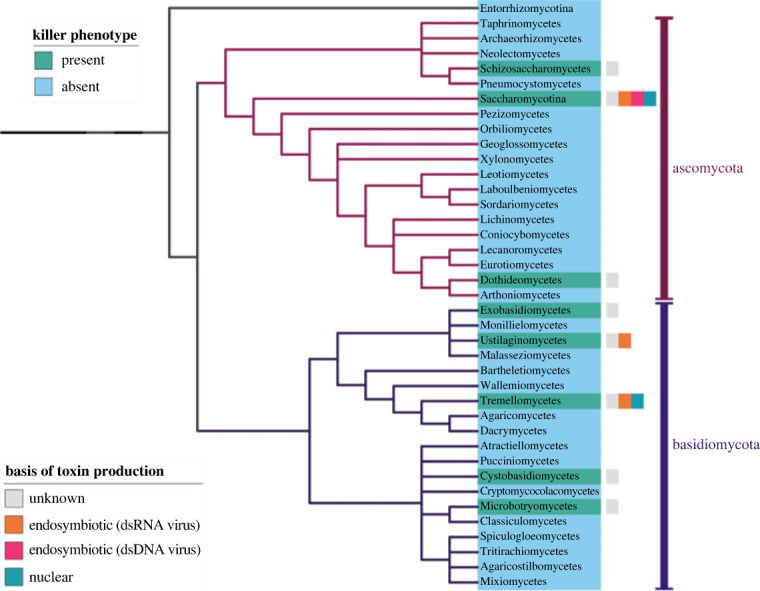
Class-level phylogenetic distribution of known killer phenotypes across the Dikarya subkingdom of fungi. The original tree was provided by Naranjo-Ortiz & Gabaldón [41], which we reduced to show the sub-phyla of Dikarya, Ascomycota (purple clade) and Basidiomycota (indigo clade) with Entorrhizomycotina as an outgroup. For most classes in possession of killer phenotypes, the genetic architecture remains unknown (grey box), whilst for others a chromosomal basis (green box), dsRNA (orange box) or dsDNA (red box) viral basis has been uncovered. Indication of the presence of killer phenotypes in a class does not mean that all species in the class have killer phenotypes.

Killer phenotypes have since been discovered in many species of the Dikarya subkingdom of fungi. Most species with killer phenotypes are ascomycetes (phylum Ascomycota), however killer phenotypes are known from more classes of Basidiomycota (figure 2; electronic supplementary material, table S1). The vast majority of killer phenotypes belong to unicellular fungi [33], and thus ‘killer yeast’ is widely used synonymously, albeit near exclusively. Not all killer phenotypes have a viral basis. Killer phenotypes can be partitioned into those whose toxin production have a chromosomal (nuclear) basis and those with an endosymbiotic basis (electronic supplementary material, table S1). Endosymbiont-encoded killer phenotypes can also be subdivided by genetic material into the aforementioned dsRNA-based killer phenotypes and their dsDNA equivalents (often called virus-like elements). Where the genetic basis of toxin production has been determined, a chromosomal basis is most frequent, while dsRNA-based killer systems are the most common of those with an endosymbiotic basis (electronic supplementary material, table S1). The genetic basis of toxin production however remains unknown for most species with killer phenotypes, including for some that are relatively well studied (electronic supplementary material, table S1); this certainly undermines attempts to explore the macroevolutionary origins of this phenomenon. Whether killer phenotypes have been discovered in a genus is a function of how well studied the genus is [42], indicating that more will be uncovered if attempts are made to do so.

### (c) The function of fungal killer phenotypes

Killer phenotypes require an expression of genes that encode proteinaceous modules with toxic properties [16]. As killer phenotypes can directly inhibit the growth of their competitors, they are often considered strategies for interference competition (figure 1; [43]). Depending on the killer system in question, killer toxins can either eliminate both allospecifics and conspecifics, or solely the former [27,42]. Interference competition is the most credible function of killer phenotypes that exclusively eliminate allospecifics. Interference competition can free up resources that are otherwise shared and depleted [30,44,45]. Under this model, killer phenotypes are maintained for the function they provide to the fungal host, whereby the fitness of the toxin-encoding genetic elements are aligned with the host's background genomes.

An alternative model for the evolution of killer phenotypes are toxin–antitoxin systems, a phenomenon well documented in bacteria [46–49]. Clonal variants that break the association with their toxin-encoding genetic elements also lose their immunity and are killed [27,34,50,51]. Toxin–antitoxin systems are believed to be selected for from the perspective of toxin-encoding genetic elements, as they prevent the host from losing them. Indeed, toxin production and self-immunity of many killer phenotypes are linked through the same gene or cytoplasmic genetic element, thus giving some credence to this hypothesis [52–54].

These models of killer phenotype function and maintenance are not necessarily mutually exclusive. The interference competition model excels at explaining how a killer phenotype would invade a community or prevent invasions from distantly related competitors, while the toxin–antitoxin model explains how a toxin-producing monoculture is protected against mutants that do not accommodate the costs of maintaining toxin-encoding genetic elements.

### (d) The ecology and evolution of fungal killer phenotypes are understudied

**Figure 3.**
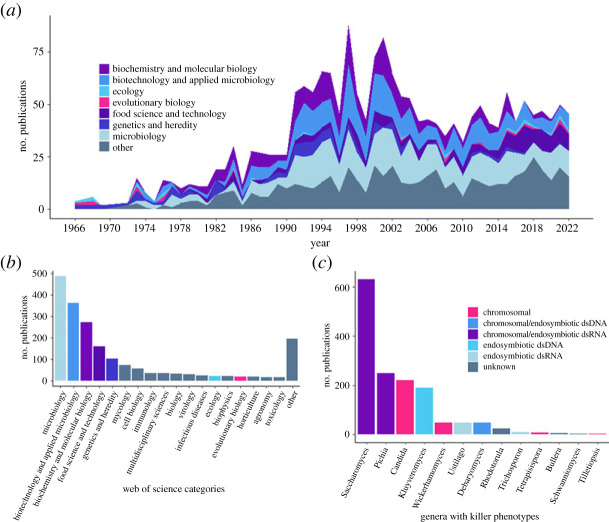
Summary of published scientific literature on the fungal killer phenomenon. We conducted a quantitative literature survey with the search tools of the Web of Science (WOS; clarivate analytics). We used the term ‘killer yeast’ for 2A and 2B, finding 1194 papers between 1966 and 2022 after manual filtering to remove irrelevant publications that were picked up by the search criteria. (*a*) The graph shows the number of publications per year within a WOS designated category, with the top five categories and ecology and evolutionary biology indicated by colour. (*b*) The most frequently reported WOS categories for publications concerning ‘killer yeast’ are provided with the number of times they are assigned to publications, with the same categories as in 2A being highlighted. Multiple assignments are possible for 2A and 2B, totalling at 1978 assignments. (*c*) The total number of publications exploring the killer phenotypes present in a selection of genera, colour coded to indicate the genetic basis of their toxins. We used the terms ‘killer’, ‘toxin’ and the name of the genera (e.g. *Saccharomyces*).

Fungal killer phenotypes were discovered 60 years ago, in which time major advances in understanding this phenomenon have been made. Where does our understanding of this phenomenon come from? To address this question, we conducted a literature survey using the Web of Science database (WOS; figure 3). Microbiology was the most frequent categorization of killer yeast publications (24.5% of all categories mentioned). Biotechnology and applied microbiology were second amongst publication categories (18.2%), research typically investigating the use of killer toxins for suppressing crop pests and opportunistic pathogens. Most basic research has been directed towards the biochemical (13.7%) and genetic (5.3%) mechanisms underlying the internal (endosymbiotic) and external (competitive) interactions involved in killing (figure 3*b*). Biochemical and cellular-level research have provided insights into toxin action and immunity [30,55–57], toxin size and structure [58,59], replication, transcription and translation of endosymbionts [60,61], as well as isolating a number of host genes that endosymbionts depend upon [62–64].

The basic organismal biology of killer phenotypes has rarely been explored (figure 3*a,b*). From 1966 to 2022, there were on average less than a single publication per year categorized by WOS as belonging to the ecology or evolutionary biology disciplines. Publications concerning *Saccharomyces* were 34.4% of the pooled publications for focal genera, 2.5 times as many publications as that of the second most studied taxa, *Pichia* (figure 3*c*). It is apparent that the basic evolutionary ecology of fungal killer phenotypes is understudied, and that there are many underexplored killer systems to do so with. Hereafter, we address our understanding of the ecology and evolution of this phenomenon to demonstrate its relevance to the killer phenotype research community and beyond.

## 3. Fungal killer phenotypes in populations and communities

### (a) Abiotic controls on killing and its effectiveness

Killer phenotypes eliminate toxin-sensitive competitors. Toxin production is typically activated by highly specific environmental cues such as pH, temperature or salt concentration [20,27]. For killer phenotypes to be beneficial to fungi, the timing of toxin production should coincide with when it can be effective, which would in turn require a trigger linking an environmental stimuli to expression of toxin-encoding genes. Abiotic and biotic parameters are often coupled [65,66], whereby changes in abiotic conditions can induce changes in community composition, and vice versa; it seems plausible that toxin production is linked to species turnover in competitive environments through abiotic conditions. However, much further research is required to confirm this link.

Moreover, toxins are released into the external environment in a diffusive fashion, being diluted in the process. Toxins can diffuse and eliminate competitors over a centimetre away on agar plates [67,68]. Natural environments are far more heterogeneous, which may impede the distances at which toxins are effective. Furthermore, the dilution process reduces the effectiveness of killer toxins. Effective concentrations of toxins have only been found to occur when producers congregate [69], which may be most problematic for killer phenotypes in aquatic environments [70]. Where diffusibility is low, killer toxins may not extend far beyond clonal siblings. However, detailed assessments of toxin activity in natural or semi-natural environments have not been conducted.

### (b) Is killing part of a cooperative strategy in yeast?

**Figure 4.**
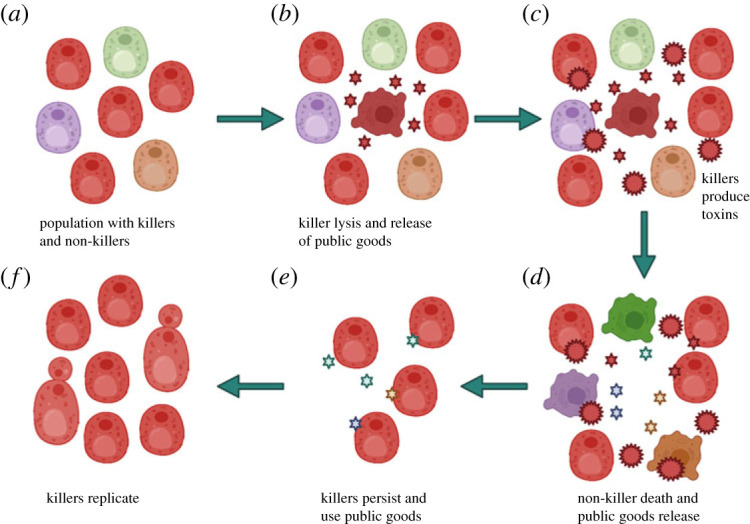
A basic schematic of how killer toxins may function in a broader cooperative strategy. (*a*) Clonally reproducing toxin producers cooccur with unrelated strains. (*b*) A fraction of the killer population undergoes cell lysis and releases public goods. (*c*) Release of public goods or a coinciding stimulus activates toxin production in the remaining killers. (*d*) Toxins are used to eliminate unrelated competitors which also release public goods. (*e*) All the public goods of competitors are shared among killers, (*f*) whom use the resources to replicate and persist. The figure was made with the BioRender graphics software.

Toxin-sensitive competitors must be in the vicinity for toxin production to be beneficial. Killer yeast fitness is greatest when their competitors are toxin-sensitive [71]. Clonal reproduction of killer yeast in structured environments results in clonal siblings all having the same killer phenotype and corresponding self-immunity. As toxin effectiveness against toxin-sensitive competitors is density-dependent and requires congregations of killer yeast [69], toxins are unlikely to be used as a tool for invading occupied niches. It is more tenable that toxins are used to prevent invasions from other unrelated taxa. Many yeast and yeast-like fungi demonstrate cooperative traits, ranging from facultative multicellularity to public goods production and sharing [72–74]. High relatedness is typically a requirement for cooperation [75–78]. Cooperation is evolutionarily unstable in the absence of mechanisms that counteract the selective incentive to cheat [79–81]. Antagonism is a known strategy of defector control in bacteria, and may apply to fungi [82]. As fungi with killer phenotypes cannot kill clonal cohabiters, toxin production may be used to filter unrelated competitors that may otherwise use public goods without any form of reciprocation. Though some unrelated competitors may have non-homologous immunity, killer toxins may shrink the pool of defectors. Rivero *et al.* [83] reported that in high cell density cultures of *S. cerevisiae*, altruistic fractions of killer populations undergo cell lysis and release Hsp12p for the public good, and the remaining killer population then release toxins to eliminate distantly related toxin-sensitive competitors in order to direct resources towards closely related kin ([83]; figure 4). This result, while standalone, indicates that killer phenotypes may function alongside other population-level social processes. This may not apply to all killer systems as not all yeast and yeast-like fungi are likely to be so cooperative. Considering the potential ancestral relatedness of many killer phenotypes, their functions may have diversified in accordance with the ecology of the hosts that wield them.

### (c) Do killer phenotypes drive coevolutionary dynamics with host competitors?

The elimination of toxin-sensitive competitors reduces genetic and functional diversity in the populations and communities of killer yeast. Some species use toxins to eliminate members of the same species while others can eliminate individuals from distant species [42], including bacteria [84–86]. The consequences of genotype or species extinction for population viability, community structure and ecosystem function depend on the degree of functional redundancy in the system. Reductions in genetic diversity can hinder population adaptability in ever changing environments [87–89]. Elimination of keystone species would however have the greatest impact on communities, though studies have not linked toxin-sensitive competitors to their ecological functions. Where toxin-sensitive competitors outnumber toxin-resistant competitors, killer phenotypes may radically change community composition and structure despite toxin-sensitive competitors not providing keystone functions. Connectedness and closeness centrality are often used as indicators of a species' community-level function [90,91], and have previously been used to identify keystone species in microbial communities [92]. Who kills whom, and how, of naturally co-occurring yeast strains and species remains a largely unexplored topic, thus the knock-on effects of killer activity on communities remain unknown.

Killer phenotypes can select for toxin resistance in their competitors [93]. Toxin resistance should emerge rapidly if resistance polymorphism is present. Resistance variation exists within and between species [43]. Resistance can be fixed in some species but not in congenerics, demonstrating different coevolutionary histories with antagonistic allelopathy [94]. The genetic basis of resistance has been uncovered on few occasions. The most complete assessment of resistance found that the *KTD1* gene, and a number of its alleles, provide different levels of protection against the *S. cerevisae* K28 toxin [95]. Resistance tends to be towards toxins of cooccurring killer strain, as opposed to formerly unencountered killer phenotypes, suggesting killer-mediated evolution in competitors [43]. If resistance is fixed among non-killer competitors, carrying a killer phenotype is likely to become costly [71]. While resistance has been demonstrated to evolve, there is no evidence of evolution in toxin producers to overcome resistance. The prevalence of killer phenotypes in natural populations, albeit often at low frequency [94,96–99], suggests resistance can be overcome. Toxin-resistance evolution may select upon toxin polymorphism, enabling the spread of killer phenotypes for which limited resistance is found.

### (d) Are there intransitive dynamics between killers and non-killers?

If toxin production is more energetically expensive than resistance but the latter still bears a cost, then toxin-resistant competitors may overcome toxin producers and toxin-sensitive strains may replace their toxin-resistant competitors in toxin-free environments. Intransitive fitness costs are theorized to maintain diversity in both continuous habitats with dispersal limitation and patchy habitats [100–104]. Abiotic conditions may also drive intransitive dynamics, as the occurrence of toxin producers is dependent on the stage of fermentation and its pH, which may suggest toxin producers are excluded when toxin production is inactive [43,105]; toxin-sensitive competitors may even invade the range of toxin producers when toxins are absent. The intransitive interactions of microbial fungi are expected to resemble those of bacteria [101,102,106,107], such that literature of the latter often supplements that of the former. However, it is not known whether fungal populations and communities experience intransitivity *in situ*. For example, it remains unclear whether resistance bears a strict cost, or how the rates and intensities of exploitation and interference competition intersect.

### (e) Model system for competitor coevolution within populations and communities

Competitive coevolutionary dynamics are rarely studied [28,108,109]. Killer phenotypes provide an opportunity to expand beyond the standard model systems of coevolution. Progress in understanding how killer phenotypes affect their populations and communities depends on gaining a more resolute understanding of who kills whom *in situ*, which can be accomplished *ex-situ* with a variety of different killer assay tests [16–20]. Killer phenotypes in natural populations have been detected in numerous studies [43,97,99,110] as have the relative frequencies of toxin-resistant and toxin-sensitive competitors [43,111]. However, a successful killing is a genotype×genotype×environment (GxGxE) interaction; evidence for toxin-encoding genes or toxin sensitivity in a tester strain does not equate to a killer in nature. Instead, it calls for spatio-temporal all-versus-all killer assays wherever feasible. By coupling killer assays with sequencing, insights can be made into how killer phenotypes modify genetic and species diversity, and whether antagonistic coevolution is responsible for the maintenance of genetic diversity.

## 4. The evolutionary ecology of endosymbiont-encoded killer phenotypes

### (a) The origins of the multi-viral basis of killer phenotypes

Endosymbiont-encoded killer phenotypes depend on two viral types, toxin-encoding satellite viruses and the autonomous helper viruses that they depend on for their replication and maintenance (figure 1; [27,30,112,113]). The ubiquity of a multipartite viral basis to endosymbiont-encoded killer phenotypes suggests that they may share a common origin. The dsRNA helper and satellite viruses of many Saccharomycotina yeasts have similar genetic architecture, in support of the common origin hypothesis [54,114–118]. Phylogenetic relationships between viruses across classes have not been assessed because sequence data are unavailable. The helper viruses of the various dsRNA killer systems are totiviruses (Family Totiviridae), a monophyletic group distinguished by undivided dsRNA genomes encoding coat protein and polymerase genes. Totiviruses have a long history of co-divergence with their fungal hosts as a result of almost exclusive vertical transmission [119]. Extant helper and satellite viruses of Saccharomycotina appear to be both a product of co-divergence with fungal hosts [120–122] and host switching via interspecific hybridization [117]. The origins of endosymbiont-encoded killer phenotypes are unresolved, though there is a strong link between the presence of killer phenotype-encoding dsRNA viruses and the absence of a functional RNA interference (RNAi) system in the host [123]. RNAi is an immune defence that acts to degrade foreign RNAs [124] so its absence may be a prerequisite for killer systems with a dsRNA viral genetic basis to evolve.

The origins of a dsDNA viral basis for killer phenotypes are even less well understood. dsDNA viruses were first known as linear plasmids [50,125–127]. However, they are now known to share a number of features with viral groups which are unlikely to have convergently evolved [128–131]. Of the extant dsDNA virally encoded killer systems, homology among them suggests a monophyletic origin [132]. Endosymbiotic dsDNA killer systems also have a helper-satellite viral configuration enabling toxin production; though this is certainly a case of convergent evolution, it does raise questions about the benefits of viral multipartism for toxin production in fungi.

### (b) Host-helper-satellite interactions

The fitness consequences of killer phenotypes and mycoviruses for fungi are context-dependent (figure 1; [71]). Some helper viruses have been demonstrated to detriment host fitness. For *S. cerevisiae*, uncontrolled proliferation of its helper virus results in proteostatic stress [133]. Unlike with bacterial phages, evidence is lacking for mycovirally induced cell lysis and subsequent horizontal transmission [134]; vertical transmission induces fidelity feedbacks in which exploitation of the host equally detriments the endosymbionts [79,135]. Some fungi have evolved strategies to mitigate against the costs of their toxin-encoding mycoviruses. Xrn1p, which is involved in cytoplasmic mRNA degradation, is co-opted by *Saccharomyces* to degrade its helper viruses, particularly variants it has coevolved with [122]. During sporulation when toxin production is not occurring, some yeast attenuate their viruses, and by doing so they lower the viral copy number and prevent excessive proliferation and the associated costs being passed on to progeny [136].

Hosts have restricted cellular control over their endosymbionts, as the latter do not abide by host replication cycles [137]. Hosts can take control of toxin production by incorporating toxin- and immunity-encoding genes into the nuclear genome as a prerequisite for expelling their endosymbionts [138]. Many Saccharomycotina species possess pseudogenes homologous to toxin-encoding and immunity genes [139,140]. Selection is unlikely to maintain toxin-encoding endosymbionts if genome integration is a viable alternative [141–143]; by having a chromosomal copy hosts can lose their viruses without consequences. However, this introduces host-endosymbiont conflict, and selection should favour viruses that oppose integration. Some dsDNA viruses possess high A/T content genomes that are cleaved by nuclear transcription apparatuses upon attempted incorporation of virally encoded immunity genes by hosts [144]. Though killer phenotypes may provide competitive benefits to their hosts, all the evidence points toward conflict between killer phenotype-encoding genetic elements and their hosts.

Satellite viruses do not bear equivalent costs on their fungal hosts as that of helper viruses. Satellite viruses dampen helper virus proliferation because they use the same resources, which should reduce the helper virus burden on their hosts (figure 1; [62,145]). Removal of the toxin-encoding satellite viruses resulted in fewer significant gene up/downregulations in *S. cerevisiae* than occurred upon removal of the helper viruses [120,146]. Toxin-encoding satellite viruses are typically portrayed as exploiters of helper viruses, because they use the produce of helper viruses and do not reciprocate any immediate resource or information [30,147]. However, satellite viruses provide indirect benefits to helper viruses, through the provision of immunity and toxins to the hosts that helper viruses depend on. As this is indirect, helper viruses may still evolve strategies to mitigate the satellites exploitation, but in the long-term, fungal cells with only the helper virus should be selected against.

Interactions between endosymbiont classes and host genomes determine the effectiveness of toxins. Naturally coevolved helper-satellite combinations provide the most effective toxins to their hosts [121]. Killer toxins were however tested against killer phenotype-free variants of the same strains [121], which may instead evidence a toxin–antitoxin system in which killer phenotype-encoding genetic elements are adapted to killing its host genotype. In a competitive context, these results could however indicate that host-endosymbiont interactions determine how killer toxins select for resistance in their competitors [121].

### (c) The consequences of sex for endosymbiont-encoded killer phenotypes

Horizontal transmission via cytoplasmic mixing is seemingly the only feasible way for mycoviruses to spread to new lineages [148]. Although mycoviruses can be attenuated during sporulation, they do persist in low frequencies [136]. While clonal mating events may only reduce mycovirus copy number, outcrossing provides the possibility for offspring to inherit endosymbionts from both parents, as they often do with mitochondria [147,149]. Past research found that distinct killer systems from different hosts cannot coexist together in daughter cells, having found that only one helper and satellite virus combination ever persisted [150,151]. Combinations of viruses from one parent are expected to persist due to their coadaptation from past vertical transmission, and the greater effectiveness of their toxins [121]. Mismatched viral class combinations have however been found in *S. cerevisiae* (M1 and L-Alus instead of M1 and L-A1), supposedly from past mating events [152]. These findings may suggest that toxin effectiveness is not a priority, or that selection against mismatching is weak or absent. Novel host-helper-satellite combinations may even induce intergenomic epistasis [153].

### (d) Model system for game theoretic and related questions

Virally encoded killer systems are context-dependent mutualisms with asymmetric obligations for association (figure 1). For fungi, the net cost-benefit outcome of having a virally encoded killer phenotype (figure 1*c*) is dependent on the levels of competition and resistance, and the relatedness of competitors to toxin-producers (figure 1*d*). By contrast, both helper and toxin-encoding satellite viruses are obligately dependent on their hosts (figure 1*a*), the latter's dependence being indirect through its dependence on helper viruses (figure 1*e*). Toxin-encoding satellite viruses depend entirely on helper viruses (figure 1*b*), though competitive context again determines whether helper viruses are dependent on their satellites by an indirect mutualism (figure 1*f*). These conditions provide a unique experimental platform for game theoretic questions, as does the consideration that partner choice, host sanctions, fidelity feedbacks and public goods can all be observed in natural or synthetic combinations of fungi, helper viruses and satellite viruses. Research should address how these stabilizing mechanisms interact with context dependence and dependence asymmetry in the system. However, the major goal should be to establish whether signatures of coevolutionary dynamics on killer phenotype-encoding genetic elements and their background genomes resemble those expected from antagonistic or mutualistic interactions.

## 5. The underexplored chromosomal basis of killer phenotypes

### (a) Does a chromosomal basis of toxin production improve fitness?

The norm of past fungal killer phenotype research has been to investigate the remarkable multi-viral basis of antagonistic allelopathy despite chromosomally encoded killer phenotypes being most common (figure 2; electronic supplementary material, table S1). A chromosomal basis of toxin production is usually ascertained through refutation of an endosymbiotic basis, which typically involves demonstrating that removing mycoviruses does not equate to loss of a killer phenotype [154–156]. The underlying genetics of toxin production has rarely been established for chromosomally encoded killer systems [157–159].

A translocation bias of endosymbiotic toxin-encoding genes into the nuclear genome may be partly responsible for the prevalence of chromosomal killer phenotypes [117,139]; when coupled with toxin-encoding genes that are not endosymbiotic in origin [157–159], it should come as no surprise that chromosomally encoded killer phenotypes are most common. Selection may also favour chromosomally encoded killer phenotypes as they can mitigate against the aforementioned costs of relying on endosymbionts for toxin production (§4). Polymorphism in the genetic basis of toxin production may however be maintained by fitness costs associated with chromosomally encoded killer phenotypes. Genomic integration may disrupt functional host genes, as is an occasional consequence of prophage integration into bacterial genomes [160]. Redundant toxin-encoding pseudogenes across Saccharomycotina [139] indicate that there is not selection to maintain functional killer phenotypes upon genome integration. Moreover, nuclear mutation rates are low which may undermine toxin adaptability once competitors evolve resistance. By contrast, there is selection on toxin-encoding endosymbionts to maintain themselves as being distinct from the nuclear genome (counteracting the translocation bias) [144]. Furthermore, toxin-encoding endosymbionts that maintain functional toxins should be selected for. Endosymbiont-based killer genes with their plausibly higher mutation rates [161–163] may additionally provide more opportunity for counter-adaptation once resistance emerges.

This is however all mere speculation requiring exploration. We do not know whether selection acts on the relative fitness of chromosomally encoded killer phenotypes and their virally encoded equivalents in natural environments, or anything about the proportion of chromosomally encoded killer phenotypes that have endosymbiotic origins. Certainly there are more chromosomally encoded killer phenotypes to be uncovered; genome-wide scans, as well as tetrad analysis and sequencing can be used to find them [164].

### (b) Does sex disrupt chromosomal killer phenotypes?

Asexual reproduction of fungi results in non-recombined inheritance of chromosomally encoded toxin genes. Sexual reproduction and recombination can create novel genetic diversity [165–168], including in toxin-encoding genes that are found at chiasma. Novel allelic combinations in toxin-encoding genes may enable killer toxins to overcome competitor resistance, but they may also be rendered inoperative [169,170]. If toxin-encoding genes are not present in all haplotypes, or if they are found at different loci, recombination would probably disrupt their functionality. Toxin-encoding genes could be found at different loci if they have endosymbiotic origins with independent integration events, or if they are associated with transposable elements [171]. Chromosomally encoded killer phenotypes may in turn depend on zygosity, yet the presence of heterozygosity at killer loci and consequences of such on the phenotypes expressed remain unexplored.

## 6. Fungal killer phenotypes and the mode of host reproduction

The consequences of reproduction mode for killer phenotypes remains uncharted among conducted research. A variety of reproductive strategies are available to Dikarya [172,173]. Multiple alternatives can be wielded by the same individuals, such that many are facultatively sexual and reproductive mode is determined by their ecology and population genetic structure [11,174–176]. The form of sexual reproduction can vary from selfing (homothallism) to outcrossing (heterothallism), whereby specific abiotic and biotic conditions determine which occurs. Fungal killer phenotypes thus present a remarkable system to explore how ecology and mating system interact to influence a phenotypic function.

## 7. Fungal killer phenotypes: interference competition strategy or toxin–antitoxin systems?

Throughout this review, we have discussed fungal killer phenotypes with two functions in mind: the interference competition and toxin–antitoxin system functions. The interference competition function proposes that killer phenotypes are maintained for eliminating niche-overlapping competitors and that toxin production is to the benefit of the collective genomes of the fungi [42,43]. The toxin-antitoxin system hypothesis, grounded in toxin-immunity linkage, proposes that killer phenotypes are maintained by eliminating clonal siblings that lose their killer phenotypes [138,144,177]. This hypothesis inherently posits that the fitness of killer phenotype-encoding genetic elements and their host cell's background genomes are misaligned.

There is mixed evidence for both functions. Some species are only known to eliminate allospecifics, in support of the interference competition function, while others that can eliminate conspecifics lend moderate support to a toxin–antitoxin system [42]. Under both hypotheses, there are specific targets of killer toxins. Under the toxin–antitoxin hypothesis, toxin production has evolved to kill conspecifics that have lost their killer phenotypes while the interference competition hypothesis typically predicts that killer phenotypes have evolved to eliminate competitors from the same niche [43,178]. There is however the problem of mistaking a fortuitous toxin-sensitive competitor for a target. Killer assay experiments for focal killer phenotypes exploring how relatedness and niche overlap influence toxin effectiveness could be a first step in unravelling the functions in nature.

The evolution of RNAi deficiency certainly goes against the toxin–antitoxin model (§4). RNAi could prevent the hostage-like intergenomic conflict predicted from toxin-antitoxin systems, so it is implausible that selection would act against having such a defence. However, the strongest evidence for a toxin–antitoxin system has been found in the endosymbiotic dsDNA killer systems, as opposed to the dsRNA equivalents that tend to be RNAi-deficient. Thus there is a certainly a case to be made for considering the function of each killer phenotype on a case by case basis.

As we have previously pointed out, the need for clonal congregation for effective concentrations of toxins suggests that killer phenotypes may be primarily used to eliminate distantly related competitors that would otherwise invade their populations, cheat and deplete resources (§3). This certainly resembles the toxin–antitoxin model whereby killer phenotypes eliminate mutant killer phenotype-free clones that could otherwise spread to fixation if bearing a killer phenotype is costly. The distinction between interference competition and toxin-antitoxin system models of killer phenotype function may thus be best clarified by considering how they initially evolved and in which context the phenotype is found to be beneficial.

## 8. Concluding remarks

Many fungi with killer phenotypes are ecologically, economically and medically important species; thus understanding how fungi use their killer phenotypes, and the drivers of toxin effectiveness, is fundamental to understanding how they modify their populations, communities and ecosystems. What makes this phenomenon so unique is its feasibility for addressing such diverse topics across the disciplines of ecology and evolution. Organismal approaches to the study of killer phenotypes have played a subservient function to the interest in its cell biology and applications to biotechnology. Nonetheless, a better understanding of the ecology and evolution of this phenomenon would certainly be of use to other disciplines interested in this phenomenon. Here, we have outlined some major topics in evolutionary ecology that can be addressed using this system, though they only scratch the surface. The discernibility of killing, particularly in well-studied model organisms with well-documented genetic architecture, makes it a particularly enticing phenomenon for experimental evolutionary ecology. However, the study of natural populations and communities is where the use of killer phenotypes may be most valuable. Killer phenotypes are traits whose effectiveness can be discerned *ex situ* without any great difficulty, the value of such to the study of coevolutionary dynamics is indisputable. Evident throughout our review, however, is the bias towards studying killer phenotypes in *Saccharomyces* or other Saccharomycotina taxa. Fungal killer phenotypes are found across the Dikarya, many of which remain barely studied; future research should expand beyond the usual suspects to reveal the functional phylogenetic utility of this phenomenon.

## Future issues

(i) How are the various killer phenotypes in different classes and phyla related, if at all? How many independent emergences of the fungal killer phenomenon have there been?
(ii) How do killer phenotypes contribute to the structure of populations and communities? Do they drive temporal turnover of diversity?
(iii) How does killing interact with other population-level processes, e.g. public good sharing?
(iv) What is the genomic basis of non-homologous resistance evolution? Can killers overcome resistance? Can antagonistic allelopathy drive coevolutionary dynamics?
(v) How do competitor coevolution and host-endosymbiont coevolution feedback on one another?
(vi) Do chromosomal and endosymbiont-encoded killer systems cooccur and compete? Does selection act on their relative fitness?
(vii) What are the consequences of sexual reproduction for killer phenotypes?
(viii) What are the functional origins of killer phenotypes and do they vary from species to species?

## Data Availability

Data are available in the electronic supplementary material [179].

## References

[1] Simberloff D. 1982 The status of competition theory in ecology. Ann. Zool Fenn. **19**, 241-253.

[2] Case TJ, Gilpin ME. 1974 Interference competition and niche theory. Proc. Natl Acad. Sci. USA **71**, 3073-3077. (10.1073/pnas.71.8.3073) 4528606 PMC388623

[3] Grether GF, Okamo KW. 2022 Eco-evolutionary dynamics of interference competition. Ecol. Lett. **25**, 2167-2176. (10.1111/ele.14091) 35986619

[4] Amarasekare P. 2002 Interference competition and species coexistence. Proc. R. Soc. B **269**, 2541-2550. (10.1098/rspb.2002.2181) PMC1691191 12573068

[5] Adler FR, Mosquera J. 2000 Is space necessary? Interference competition and limits to biodiversity. Ecology **81**, 3226-3232. (10.1890/0012-9658(2000)081[3226:ISNICA]2.0.CO;2)

[6] Gorter FA, Tabares-Mafla C, Kassen R, Schoustra SE. 2021 Experimental evolution of interference competition. Front. Microbiol. **12**, 613450. (10.3389/fmicb.2021.613450) 33841345 PMC8027309

[7] Chesson P. 2000 Mechanisms of maintenance of species diversity. Annu. Rev. Ecol. Syst. **31**, 343-366. (10.1146/annurev.ecolsys.31.1.343)

[8] Tilman D. 1994 Competition and biodiversity in spatially structured habitats. Ecology **75**, 2-16. (10.2307/1939377)

[9] Hastings A. 1980 Disturbance, coexistence, history, and competition for space. Theor. Popul. Biol. **18**, 363-373. (10.1016/0040-5809(80)90059-3)

[10] Thompson JN. 1994 The coevolutionary process. Chicago, IL: University of Chicago Press.

[11] Otto SP, Gerstein AC. 2006 Why have sex? The population genetics of sex and recombination. Biochem. Soc. T. **34**, 519-522. 10.1042/BST0340519 16856849

[12] Vermeij GJ. 1994 The evolutionary interaction among species - selection, escalation, and coevolution. Annu. Rev. Ecol. Syst. **25**, 219-236. (10.1146/annurev.es.25.110194.001251)

[13] Amandine C, Ebert D, Stukenbrock E, de la Vega RCR, Tiff P, Croll D, Tellier A. 2022 Unraveling coevolutionary dynamics using ecological genomics. Trends Genet. **38**, 1003-1012. (10.1016/j.tig.2022.05.008) 35715278

[14] Boddy L. 2000 Interspecific combative interactions between wood-decaying basidiomycetes. FEMS Microbiol. Ecol. **31**, 185-194. (10.1111/j.1574-6941.2000.tb00683.x) 10719199

[15] Boddy L, Hiscox J. 2016 Fungal ecology: principles and mechanisms of colonization and competition by saprotrophic fungi. Microbiol. Spectr. **4**, 4-6. (10.1128/microbiolspec.FUNK-0019-2016) 28087930

[16] Makower M, Bevan EA. 1963 The physiological basis of the killer character in yeast. In Genetics today: Proc. XIth Int. Congress of Genetics, *September 1963, The Hague, The Netherlands*. Oxford, UK: Pergamon Press.

[17] Hodgson VJ, Walker GM, Button D. 1994 A rapid colorimetric assay of killer toxin activity in yeast. FEMS Microbiol. Lett. **120**, 201-205. (10.1111/j.1574-6968.1994.tb07031.x) 8056291

[18] Magliani W, Conti S, Gerloni M, Bertolotti D, Polonelli L. 1997 Yeast killer systems. Clin. Microbiol. Rev. **10**, 369. (10.1128/CMR.10.3.369) 9227858 PMC172926

[19] Klassen R, Meinhardt F. 2002 Linear plasmids pWR1A and pWR1B of the yeast *Wingea robertsiae* are associated with a killer phenotype. Plasmid **48**, 142-148. (10.1016/S0147-619X(02)00101-4) 12383731

[20] Lopes CA, Sangorrin MP. 2010 Optimization of killer assays for yeast selection protocols. Rev. Argent. Microbiol. **42**, 298-306. 21229201 10.1590/S0325-75412010000400011

[21] Polonelli L, Archibusacci C, Sestito M, Morace G. 1983 Killer system - a simple method for differentiating *Candida albicans* strains. J. Clin. Microbiol. **17**, 774-780. (10.1128/jcm.17.5.774-780.1983) 6345575 PMC272739

[22] Schaffrath R, Breunig KD. 2000 Genetics and molecular physiology of the yeast *Kluyveromyces lactis*. Fungal Genet. Biol. **30**, 173-190. (10.1006/fgbi.2000.1221) 11035939

[23] Passoth V, Fredlund E, Druvefors UA, Schnurer J. 2006 Biotechnology, physiology and genetics of the yeast *Pichia anomala*. FEMS Yeast Res. **6**, 3-13. (10.1111/j.1567-1364.2005.00004.x) 16423066

[24] Karathia H, Vilaprinyo E, Sorribas A, Alves R. 2011 *Saccharomyces cerevisiae* as a model organism: a comparative study. PLoS ONE **6**, e16015. (10.1371/journal.pone.0016015) 21311596 PMC3032731

[25] Klassen R, Kast A, Wunsche G, Paluszynski JP, Wemhoff S, Meinhardt F. 2014 Immunity factors for two related tRNA(Gln) targeting killer toxins distinguish cognate and non-cognate toxic subunits. Curr. Genet. **60**, 213-222. (10.1007/s00294-014-0426-1) 24719080

[26] Gier S, Simon M, Gasparoni G, Khalifa S, Schulz MH, Schmitt MJ, Breinig F. 2020 Yeast viral killer toxin K1 induces specific host cell adaptions via intrinsic selection pressure. Appl. Environ. Microb. **86**, e02446-19. (10.1128/AEM.02446-19) PMC6997729 31811035

[27] Marquina D, Santos A, Peinado J. 2002 Biology of killer yeasts. Int. Microbiol. **5**, 65-71. (10.1007/s10123-002-0066-z) 12180782

[28] Abrams PA. 2000 The evolution of predator-prey interactions: theory and evidence. Annu. Rev. Ecol. Syst. **31**, 79-105. (10.1146/annurev.ecolsys.31.1.79)

[29] Gier S, Lermen M, Schmitt MJ, Breinig F. 2019 Substitution of cysteines in the yeast viral killer toxin K1 precursor reveals novel insights in heterodimer formation and immunity. Sci. Rep-UK **9**, 13127. (10.1038/s41598-019-49621-z) PMC6739482 31511600

[30] Schmitt MJ, Breinig F. 2006 Yeast viral killer toxins: lethality and self-protection. Nat. Rev. Microbiol. **4**, 212-221. (10.1038/nrmicro1347) 16489348

[31] Breinig F, Sendzik T, Eisfeld K, Schmitt MJ. 2006 Dissecting toxin immunity in virus-infected killer yeast uncovers an intrinsic strategy of self-protection. Proc. Natl Acad. Sci. USA **103**, 3810-3815. (10.1073/pnas.0510070103) 16505373 PMC1533781

[32] Schmitt MJ, Klavehn P, Wang J, Schonig I, Tipper DJ. 1996 Cell cycle studies on the mode of action of yeast K28 killer toxin. Microbiol-Sgm. **142**, 2655-2662. (10.1099/00221287-142-9-2655) 8828235

[33] Schaffrath R, Meinhardt F, Klassen R. 2018 Yeast killer toxins: fundamentals and applications. Mycota **15**, 87-118.

[34] Woods DR, Bevan EA. 1968 Studies on the nature of the killer factor produced by *Saccharomyces cerevisiae*. Microbiol. Mol. Biol. R. **51**, 115-126. 10.1099/00221287-51-1-115 5653223

[35] Berry EA, Bevan EA. 1972 A new species of double-stranded RNA from yeast. Nature **239**, 279-280. (10.1038/239279a0) 4562032

[36] Bevan EA, Somers J. 1968 Studies on behavior of killer (K) and neutral (N) cytoplasmic determinants in yeast. Heredity **23**, 476.

[37] Bevan EA, Herring AJ, Mitchell DJ. 1973 Preliminary characterization of two species of dsRNA in yeast and their relationship to the ‘killer’ character. Nature **245**, 81-86. (10.1038/245081b0) 4582762

[38] Vodkin MH, Fink GR. 1973 Nucleic-acid associated with a killer strain of yeast. Proc. Natl Acad. Sci. USA **70**, 1069-1072. (10.1073/pnas.70.4.1069) 4577789 PMC433427

[39] Mitchell DJ, Herring AJ, Bevan EA. 1976 Genetic-control of Ds-Rna virus-like particles associated with *Saccharomyces Cerevisiae* killer yeast. Heredity **37**, 129-134. (10.1038/hdy.1976.71) 783092

[40] Bostian KA, Sturgeon JA, Tipper DJ. 1980 Encapsidation of yeast killer double-stranded ribonucleic-acids - dependence of M on L. J. Bacteriol. **143**, 463-470. (10.1128/jb.143.1.463-470.1980) 6995444 PMC294272

[41] Naranjo-Ortiz MA, Gabaldon T. 2019 Fungal evolution: diversity, taxonomy and phylogeny of the Fungi. Biol. Rev. Camb. Philos. Soc. **94**, 2101-2137. (10.1111/brv.12550) 31659870 PMC6899921

[42] Boynton PJ. 2019 The ecology of killer yeasts: interference competition in natural habitats. Yeast **36**, 473-485. (10.1002/yea.3398) 31050852

[43] Starmer WT, Ganter PF, Aberdeen V, Lachance MA, Phaff HJ. 1987 The ecological role of killer yeasts in natural communities of yeasts. Can. J. Microbiol. **33**, 783-796. (10.1139/m87-134) 3690423

[44] Chao L, Levin BR. 1981 Structured habitats and the evolution of anticompetitor toxins in bacteria. Proc. Natl Acad. Sci. Biol. **78**, 6324-6328. (10.1073/pnas.78.10.6324) PMC349031 7031647

[45] Fabrizio P, Longo VD. 2003 The chronological life span of *Saccharomyces cerevisiae*. Aging Cell **2**, 73-81. (10.1046/j.1474-9728.2003.00033.x) 12882320

[46] Magnuson RD. 2007 Hypothetical functions of toxin-antitoxin systems. J. Bacteriol. **189**, 6089-6092. (10.1128/JB.00958-07) 17616596 PMC1951896

[47] Jurenas D, Fraikin N, Goormaghtigh F, Van Melderen L. 2022 Biology and evolution of bacterial toxin-antitoxin systems. Nat. Rev. Microbiol. **20**, 335-350. (10.1038/s41579-021-00661-1) 34975154

[48] Van Melderen L, De Bast MS. 2009 Bacterial toxin-antitoxin systems: more than selfish entities? PLoS Genet. **5**, e1000437. (10.1371/journal.pgen.1000437) 19325885 PMC2654758

[49] Yamaguchi Y, Park JH, Inouye M. 2011 Toxin-antitoxin systems in bacteria and archaea. Annu. Rev. Genet. **45**, 61-79. (10.1146/annurev-genet-110410-132412) 22060041

[50] Gunge N, Tamaru A, Ozawa F, Sakaguchi K. 1981 Isolation and characterization of linear deoxyribonucleic-acid plasmids from Kluyveromyces-lactis and the plasmid-associated killer character. J. Bacteriol. **145**, 382-390. (10.1128/jb.145.1.382-390.1981) 6257636 PMC217283

[51] Worsham PL, Bolen PL. 1990 Killer toxin production in *Pichia acaciae* is associated with linear DNA plasmids. Curr. Genet. **18**, 77-80. (10.1007/BF00321119) 2245477

[52] Paluszynski JP, Klassen R, Meinhardt F. 2007 *Pichia acaciae* killer system: genetic analysis of toxin immunity. Appl. Environ. Microb. **73**, 4373-4378. (10.1128/AEM.00271-07) PMC1932769 17483256

[53] Zhu YS, Kane J, Zhang XY, Zhang M, Tipper DJ. 1993 Role of the gamma-component of preprotoxin in expression of the yeast K(1) killer phenotype. Yeast **9**, 251-266. (10.1002/yea.320090305) 8488726

[54] Ramirez M, Velazquez R, Lopez-Pineiro A, Naranjo B, Roig F, Llorens C. 2017 New insights into the genome organization of yeast killer viruses based on ‘Atypical’ killer strains characterized by high-throughput sequencing. Toxins (Basel) **9**, 292. (10.3390/toxins9090292) 28925975 PMC5618225

[55] Becker B, Schmitt MJ. 2017 Yeast killer toxin K28: biology and unique strategy of host cell intoxication and killing. Toxins **9**, 333. (10.3390/toxins9100333) 29053588 PMC5666379

[56] Bussey H, Sacks W, Galley D, Saville D. 1982 Yeast killer plasmid mutations affecting toxin secretion and activity and toxin immunity function. Mol. Cell. Biol. **2**, 346-354. 7050670 10.1128/mcb.2.4.346-354.1982 PMC369798

[57] Liu GL, Chi Z, Wang GY, Wang ZP, Li Y, Chi ZM. 2015 Yeast killer toxins, molecular mechanisms of their action and their applications. Crit. Rev. Biotechnol. **35**, 222-234. (10.3109/07388551.2013.833582) 24102112

[58] Palfree RGE, Bussey H. 1979 Yeast killer toxin: purification and characterization of the protein toxin from *Saccharomyces cerevisiae*. Eur. J. Biochem. **93**, 487-493. (10.1111/j.1432-1033.1979.tb12847.x) 33806

[59] Park CM, Banerjee N, Koltin Y, Bruenn JA. 1996 The *Ustilago maydis* virally encoded KP1 killer toxin. Mol. Microbiol. **20**, 957-963. (10.1111/j.1365-2958.1996.tb02537.x) 8809749

[60] Dinman JD, Wickner RB. 1992 Ribosomal frameshifting efficiency and gag gag-pol ratio are critical for yeast M(1) double-stranded-Rna virus propagation. J. Virol. **66**, 3669-3676. (10.1128/jvi.66.6.3669-3676.1992) 1583726 PMC241150

[61] Wickner RB, Leibowitz MJ. 1979 Mak mutants of yeast - mapping and characterization. J. Bacteriol. **140**, 154-160. (10.1128/jb.140.1.154-160.1979) 387719 PMC216791

[62] Ball SG, Tirtiaux C, Wickner RB. 1984 Genetic control of L-a and L-(Bc) dsRNA copy number in killer systems of *Saccharomyces cerevisiae*. Genetics **107**, 199-217. (10.1093/genetics/107.2.199) 17246214 PMC1202319

[63] Ohtake Y, Wickner RB. 1995 Yeast virus propagation depends critically on free 60 s ribosomal-subunit concentration. Mol. Cell. Biol. **15**, 2772-2781. (10.1128/MCB.15.5.2772) 7739558 PMC230508

[64] Wickner RB, Leibowitz MJ. 1976 Two chromosomal genes required for killing expression in killer strains of *Saccharomyces cerevisiae*. Genetics **82**, 429-442. (10.1093/genetics/82.3.429) 773743 PMC1213465

[65] Dunson WA, Travis J. 1991 The role of abiotic factors in community organization. Am. Nat. **138**, 1067-1091. (10.1086/285270)

[66] Singh BK, Dawson LA, Macdonald CA, Buckland SM. 2009 Impact of biotic and abiotic interaction on soil microbial communities and functions: a field study. Appl. Soil Ecol. **41**, 239-248. (10.1016/j.apsoil.2008.10.003)

[67] Banjara N, Nickerson KW, Suhr MJ, Hallen-Adams HE. 2016 Killer toxin from several food-derived *Debaryomyces hansenii* strains effective against pathogenic Candida yeasts. Int. J. Food Microbiol. **222**, 23-29. (10.1016/j.ijfoodmicro.2016.01.016) 26828815

[68] Tan CM, Wang L, Xue Y, Lin S, Yu G, Yang SL. 2018 Purification and molecular characterization of a *Metschnikowia saccharicola* killer toxin lethal to a crab pathogenic yeast. FEMS Microbiol. Lett. **365**, fny038. 10.1093/femsle/fny038 29462299

[69] Greig D, Travisano M. 2008 Density-dependent effects on allelopathic interactions in yeast. Evolution **62**, 521-527. (10.1111/j.1558-5646.2007.00292.x) 17983463

[70] Wang X, Chi Z, Yue L, Li J. 2007 Purification and characterization of killer toxin from a marine yeast *Pichia anomala* YF07b against the pathogenic yeast in crab. Curr. Microbiol. **55**, 396-401. (10.1007/s00284-007-9010-y) 17687604

[71] Pintar J, Starmer WT. 2003 The costs and benefits of killer toxin production by the yeast *Pichia kluyveri*. Anton Leeuw Int. J. G. **83**, 89-97. 10.1023/a:0000000089097 12755485

[72] Bachmann H, Fischlechner M, Rabbers I, Barfa N, dos Santos FB, Molenaar D, Teusink B. 2013 Availability of public goods shapes the evolution of competing metabolic strategies. Proc. Natl Acad. Sci. USA **110**, 14 302-14 307. (10.1073/pnas.1308523110) PMC3761572 23940318

[73] Fisher RM, Regenberg B. 2019 Multicellular group formation in *Saccharomyces cerevisiae*. Proc. R. Soc. B **286**, 20191098. 10.1098/rspb.2019.1098 PMC6742993 31480977

[74] Maclean RC, Brandon C. 2008 Stable public goods cooperation and dynamic social interactions in yeast. J. Evol. Biol. **21**, 1836-1843. (10.1111/j.1420-9101.2008.01579.x) 18643862

[75] Bastiaans E, Debets AJM, Aanen DK. 2016 Experimental evolution reveals that high relatedness protects multicellular cooperation from cheaters. Nat. Commun. **7**, 11435. (10.1038/ncomms11435) 27139112 PMC4857390

[76] Gilbert OM, Foster KR, Mehdiabadi NJ, Strassmann JE, Queller DC. 2007 High relatedness maintains multicellular cooperation in a social amoeba by controlling cheater mutants. Proc. Natl Acad. Sci. USA **104**, 8913-8917. (10.1073/pnas.0702723104) 17496139 PMC1885602

[77] Griffin AS, West SA, Buckling A. 2004 Cooperation and competition in pathogenic bacteria. Nature **430**, 1024-1027. (10.1038/nature02744) 15329720

[78] West SA, Buckling A. 2003 Cooperation, virulence and siderophore production in bacterial parasites. Proc. R. Soc. B **270**, 37-44. (10.1098/rspb.2002.2209) PMC1691207 12590769

[79] Foster KR, Kokko H. 2006 Cheating can stabilize cooperation in mutualisms. Proc. R. Soc. B **273**, 2233-2239. 10.1098/rspb.2006.3571 PMC1635526 16901844

[80] Frederickson ME. 2013 Rethinking mutualism stability: cheaters and the evolution of sanctions. Q. Rev. Biol. **88**, 269-295. (10.1086/673757) 24552098

[81] Smith JM. 1982 Evolution and the theory of games. Cambridge, UK: Cambridge University Press.

[82] Jousset A, Eisenhauer N, Materne E, Scheu S. 2013 Evolutionary history predicts the stability of cooperation in microbial communities. Nat. Commun. **4**, 2573. (10.1038/ncomms3573) 24113642

[83] Rivero D, Berna L, Stefanini I, Baruffini E, Bergerat A, Csikasz-Nagy A, De Filippo C, Cavalieri D. 2015 Hsp12p and PAU genes are involved in ecological interactions between natural yeast strains. Environ. Microbiol. **17**, 3069-3081. (10.1111/1462-2920.12950) 26079802

[84] Polonelli L, Morace G. 1986 Reevaluation of the yeast killer phenomenon. J. Clin. Microbiol. **24**, 866-869. (10.1128/jcm.24.5.866-869.1986) 3771773 PMC269048

[85] Meneghin MC, Reis VR, Ceccato-Antonini SR. 2010 Inhibition of bacteria contaminating alcoholic fermentations by killer yeasts. Braz. Arch. Biol. Technol. **53**, 1043-1050. (10.1590/S1516-89132010000500006)

[86] Bajaj BK, Raina S, Singh S. 2013 Killer toxin from a novel killer yeast *Pichia kudriavzevii* RY55 with idiosyncratic antibacterial activity. J. Basic Microbiol. **53**, 645-656. (10.1002/jobm.201200187) 22961241

[87] Arber W. 2000 Genetic variation: molecular mechanisms and impact on microbial evolution. FEMS Microbiol. Rev. **24**, 1-7. (10.1111/j.1574-6976.2000.tb00529.x) 10640595

[88] Booy G, Hendriks RJJ, Smulders MJM, Van Groenendael JM, Vosman B. 2000 Genetic diversity and the survival of populations. Plant Biol. **2**, 379-395. (10.1055/s-2000-5958)

[89] Hermisson J, Pennings PS. 2005 Soft sweeps: molecular population genetics of adaptation from standing genetic variation. Genetics **169**, 2335-2352. (10.1534/genetics.104.036947) 15716498 PMC1449620

[90] Gonzalez AMM, Dalsgaard B, Olesen JM. 2010 Centrality measures and the importance of generalist species in pollination networks. Ecol. Complex **7**, 36-43. (10.1016/j.ecocom.2009.03.008)

[91] Lai SM, Liu WC, Jordan F. 2012 On the centrality and uniqueness of species from the network perspective. Biol. Lett. **8**, 570-573. (10.1098/rsbl.2011.1167) 22357938 PMC3391439

[92] Karimi B, Maron PA, Boure NCP, Bernard N, Gilbert D, Ranjard L. 2017 Microbial diversity and ecological networks as indicators of environmental quality. Environ. Chem. Lett. **15**, 265-281. (10.1007/s10311-017-0614-6)

[93] Pieczynska MD, Wloch-Salamon D, Korona R, de Visser JAGM. 2016 Rapid multiple-level coevolution in experimental populations of yeast killer and nonkiller strains. Evolution **70**, 1342-1353. (10.1111/evo.12945) 27168531

[94] Chang SL, Leu JY, Chang TH. 2015 A population study of killer viruses reveals different evolutionary histories of two closely related *Saccharomyces* sensu stricto yeasts. Mol. Ecol. **24**, 4312-4322. (10.1111/mec.13310) 26179470

[95] Andreev I, Laidlaw KME, Giovanetti SM, Urtecho G, Shriner D, Bloom JS, MacDonald C, Sadhu MJ. 2023 Discovery of a rapidly evolving yeast defense factor, KTD1, against the secreted killer toxin K28. Proc. Natl Acad. Sci. USA **120**, e2217194120. 36800387 10.1073/pnas.2217194120 PMC9974470

[96] Carreiro SC, Pagnocca FC, Bacci M, Bueno OC, Hebling MJA, Middelhoven WJ. 2002 Occurrence of killer yeasts in leaf-cutting ant nests. Folia Microbiol. **47**, 259-262. (10.1007/BF02817648) 12099266

[97] Nakayashiki T, Kurtzman CP, Edskes HK, Wickner RB. 2005 Yeast prions [URE3] and [PSI + ] are diseases. Proc. Natl Acad. Sci. USA **102**, 10 575-10 580. (10.1073/pnas.0504882102) PMC1180808 16024723

[98] Perez MF, Contreras L, Garnica NM, Fernandez-Zenoff MV, Farias ME, Sepulveda M, Ramallo J, Dib JR. 2016 Native killer yeasts as biocontrol agents of postharvest fungal diseases in lemons. PLoS ONE **11**, e0165590. (10.1371/journal.pone.0165590) 27792761 PMC5085023

[99] Philiskirk G, Young TW. 1975 The occurrence of killer character in yeasts of various genera. Antonie Van Leeuwenhoek. **41**, 147-151. (10.1007/BF02565046) 239627

[100] Czaran TL, Hoekstra RF. 2003 Killer-sensitive coexistence in metapopulations of micro-organisms. Proc. R. Soc. B **270**, 1373-1378. (10.1098/rspb.2003.2338) PMC1691387 12965028

[101] Czaran TL, Hoekstra RF, Pagie L. 2002 Chemical warfare between microbes promotes biodiversity. Proc. Natl Acad. Sci. USA **99**, 786-790. (10.1073/pnas.012399899) 11792831 PMC117383

[102] Kerr B, Riley MA, Feldman MW, Bohannan BJM. 2002 Local dispersal promotes biodiversity in a real-life game of rock-paper-scissors. Nature **418**, 171-174. (10.1038/nature00823) 12110887

[103] Laird RA, Schamp BS. 2006 Competitive intransitivity promotes species coexistence. Am. Nat. **168**, 182-193. (10.1086/506259) 16874628

[104] Sinclair RM. 2014 Persistence in the shadow of killers. Front. Microbiol. **5**, 342. 25071753 10.3389/fmicb.2014.00342 PMC4095038

[105] Morais PB, Martins MB, Klaczko LB, Mendoncahagler LC, Hagler AN. 1995 Yeast succession in the Amazon fruit *Parahancornia amapa* as resource partitioning among Drosophila Spp. Appl. Environ. Microb. **61**, 4251-4257. (10.1128/aem.61.12.4251-4257.1995) PMC167736 8534092

[106] Narisawa N, Haruta S, Arai H, Ishii M, Igarashi Y. 2008 Coexistence of antibiotic-producing and antibiotic-sensitive bacteria in biofilms is mediated by resistant bacteria. Appl. Environ. Microb. **74**, 3887-3894. (10.1128/AEM.02497-07) PMC2446560 18441106

[107] Reichenbach T, Mobilia M, Frey E. 2007 Mobility promotes and jeopardizes biodiversity in rock-paper-scissors games. Nature **448**, 1046-1049. (10.1038/nature06095) 17728757

[108] Gandon S, Buckling A, Decaestecker E, Day T. 2008 Host-parasite coevolution and patterns of adaptation across time and space. J. Evol. Biol. **21**, 1861-1866. (10.1111/j.1420-9101.2008.01598.x) 18717749

[109] Maron JL, Agrawal AA, Schemske DW. 2019 Plant-herbivore coevolution and plant speciation. Ecology **100**, e02704. 30916391 10.1002/ecy.2704

[110] Pieczynska MD, de Visser JAGM, Korona R. 2013 Incidence of symbiotic dsRNA ‘killer’ viruses in wild and domesticated yeast. FEMS Yeast Res. **13**, 856-859. (10.1111/1567-1364.12086) 24028530

[111] Boynton PJ, Wloch-Salamon D, Landermann D, Stukenbrock EH. 2021 Forest *Saccharomyces* *paradoxus* are robust to seasonal biotic and abiotic changes. Ecol. Evol. **11**, 6604-6619. (10.1002/ece3.7515) 34141244 PMC8207440

[112] Fukuhara H. 1995 Linear DNA plasmids of yeasts. FEMS Microbiol. Lett. **131**, 1-9. (10.1111/j.1574-6968.1995.tb07745.x) 7557303

[113] Satwika D, Klassen R, Meinhardt F. 2012 Anticodon nuclease encoding virus-like elements in yeast. Appl. Microbiol. Biot. **96**, 345-356. (10.1007/s00253-012-4349-9) 22899498

[114] Ramirez M, Velazquez R, Maqueda M, Martinez A. 2020 Genome organization of a new double-stranded RNA LA helper virus from wine *Torulaspora delbrueckii* killer yeast as compared with its *Saccharomyces* counterparts. Front. Microbiol. **11**, 593846. (10.3389/fmicb.2020.593846) 33324373 PMC7721687

[115] Ramirez M, Velazquez R, Lopez-Pineiro A, Martinez A. 2021 Genome features of a new double-stranded RNA helper virus (LBCbarr) from wine *Torulaspora delbrueckii* killer strains. Int. J. Mol. Sci. **22**, 13492. (10.3390/ijms222413492) 34948288 PMC8709356

[116] Rodriguez-Cousino N, Esteban R. 2017 Relationships and evolution of double-stranded RNA totiviruses of yeasts inferred from analysis of L-A-2 and L-BC variants in wine yeast strain populations. Appl. Environ. Microbiol. **83**, e02991-16. (10.1128/AEM.02991-16) 27940540 PMC5288835

[117] Fredericks LR et al. 2021 The species-specific acquisition and diversification of a K1-like family of killer toxins in budding yeasts of the Saccharomycotina. PLoS Genet. **17**, e1009341. (10.1371/journal.pgen.1009341) 33539346 PMC7888664

[118] Vepstaite-Monstavice I, Luksa J, Konovalovas A, Ezerskyte D, Staneviciene R, Strazdaite-Zieliene Z, Serva S, Servienė E. 2018 *Saccharomyces paradoxus* K66 killer system evidences expanded assortment of helper and satellite viruses. Viruses-Basel **10**, 564. (10.3390/v10100564) PMC6213463 30332789

[119] Goker M, Scheuner C, Klenk HP, Stielow JB, Menzel W. 2011 Codivergence of mycoviruses with their hosts. PLoS ONE **6**, e22252. (10.1371/journal.pone.0022252) 21829452 PMC3146478

[120] McBride RC, Boucher N, Park DS, Turner PE, Townsend JP. 2013 Yeast response to LA virus indicates coadapted global gene expression during mycoviral infection. FEMS Yeast Res. **13**, 162-179. (10.1111/1567-1364.12019) 23122216

[121] Pieczynska MD, Korona R, de Visser JAGM. 2017 Experimental tests of host-virus coevolution in natural killer yeast strains. J. Evol. Biol. **30**, 773-781. (10.1111/jeb.13044) 28117504

[122] Rowley PA, Ho B, Bushong S, Johnson A, Sawyer SL. 2016 XRN1 is a species-specific virus restriction factor in yeasts. PLoS Pathog. **12**, e1005890. (10.1371/journal.ppat.1005890) 27711183 PMC5053509

[123] Drinnenberg IA, Fink GR, Bartel DP. 2011 Compatibility with killer explains the rise of RNAi-deficient fungi. Science **333**, 1592. (10.1126/science.1209575) 21921191 PMC3790311

[124] Billmyre RB, Calo S, Feretzaki M, Wang XY, Heitman J. 2013 RNAi function, diversity, and loss in the fungal kingdom. Chromosome Res. **21**, 561-572. (10.1007/s10577-013-9388-2) 24173579 PMC3874831

[125] Sor F, Fukuhara H. 1985 Structure of a linear plasmid of the yeast *Kluyveromyces lactis* - compact organization of the killer genome. Curr. Genet. **9**, 147-155. (10.1007/BF00436963)

[126] Hishinuma F, Nakamura K, Hirai K, Nishizawa R, Gunge N, Maeda T. 1984 Cloning and nucleotide-sequences of the linear DNA killer plasmids from yeast. Nucleic Acids Res. **12**, 7581-7597. (10.1093/nar/12.19.7581) 6387625 PMC320182

[127] Gunge N, Murata K, Sakaguchi K. 1982 Transformation of *Saccharomyces cerevisiae* with linear DNA killer plasmids from *Kluyveromyces lactis*. J. Bacteriol. **151**, 462-464. (10.1128/jb.151.1.462-464.1982) 7045080 PMC220260

[128] Stark MJR, Boyd A, Mileham AJ, Romanos MA. 1990 The plasmid-encoded killer system of *Kluyveromyces lactis* - a review. Yeast **6**, 1-29. (10.1002/yea.320060102) 2180235

[129] Rohe M, Schrunder J, Tudzynski P, Meinhardt F. 1992 Phylogenetic-relationships of linear, protein-primed replicating genomes. Curr. Genet. **21**, 173-176. (10.1007/BF00318478) 1568258

[130] Sykora M, Pospisek M, Novak J, Mrvova S, Krasny L, Vopalensky V. 2018 Transcription apparatus of the yeast virus-like elements: architecture, function, and evolutionary origin. PLoS Pathog. **14**, e1007377. (10.1371/journal.ppat.1007377) 30346988 PMC6211774

[131] Larsen M, Gunge N, Meinhardt F. 1998 *Kluyveromyces lactis* killer plasmid pGKL2: evidence for a viral-like capping enzyme encoded by ORF3. Plasmid **40**, 243-246. (10.1006/plas.1998.1367) 9806862

[132] Meinhardt F, Schaffrath R. 2013 Extranuclear inheritance: cytoplasmic linear double-stranded DNA killer elements of the dairy yeast *Kluyveromyces lactis*. In Progress in Botany, pp. 62: Berlin, Germany: Springer.

[133] Chau S, Gao J, Diao AJ, Cao SB, Azhieh A, Davidson AR, Meneghini MD. 2023 Diverse yeast antiviral systems prevent lethal pathogenesis caused by the LA mycovirus. Proc. Natl Acad. Sci. USA **120**, e2208695120. (10.1073/pnas.2208695120) 36888656 PMC10089162

[134] Lerer V, Shlezinger N. 2022 Inseparable companions: fungal viruses as regulators of fungal fitness and host adaptation. Front. Cell Infect. Microbiol. **12**, 1020608. (10.3389/fcimb.2022.1020608) 36310864 PMC9606465

[135] Archetti M, Scheuring I, Hoffman M, Frederickson ME, Pierce NE, Yu DW. 2011 Economic game theory for mutualism and cooperation. Ecol. Lett. **14**, 1300-1312. (10.1111/j.1461-0248.2011.01697.x) 22011186

[136] Gao J, Chau S, Chowdhury F, Zhou T, Hossain S, McQuibban GA, Meneghini MD. 2019 Meiotic viral attenuation through an ancestral apoptotic pathway. Proc. Natl Acad. Sci. USA **116**, 16 454-16 462. (10.1073/pnas.1900751116) PMC6697816 31266891

[137] Lynch M, Marinov GK. 2015 The bioenergetic costs of a gene. Proc. Natl Acad. Sci. USA **112**, 15 690-5. (10.1073/pnas.1514974112) PMC4697398 26575626

[138] Wickner RB, Edskes HK. 2015 Yeast killer elements hold their hosts hostage. PLoS Genet. **11**, e1005139. (10.1371/journal.pgen.1005139) 25973796 PMC4431855

[139] Frank AC, Wolfe KH. 2009 Evolutionary capture of viral and plasmid DNA by yeast nuclear chromosomes. Eukaryot. Cell **8**, 1521-1531. (10.1128/EC.00110-09) 19666779 PMC2756859

[140] Satwika D, Klassen R, Meinhardt F. 2012 Repeated capture of a cytoplasmic linear plasmid by the host nucleus in *Debaryomyces hansenii*. Yeast **29**, 145-154. (10.1002/yea.2893) 22434608

[141] Husnik F, Keeling PJ. 2019 The fate of obligate endosymbionts: reduction, integration, or extinction. Curr. Opin. Genet. Dev. **58**, 1-8. (10.1016/j.gde.2019.07.014) 31470232

[142] Kelly S. 2021 The economics of organellar gene loss and endosymbiotic gene transfer. Genome Biol. **22**, 1-22. (10.1186/s13059-021-02567-w) 34930424 PMC8686548

[143] Wernegreen JJ. 2012 Endosymbiosis. Curr. Biol. **22**, R555-RR61. (10.1016/j.cub.2012.06.010) 22835786

[144] Kast A, Voges R, Schroth M, Schaffrath R, Klassen R, Meinhardt F. 2015 Autoselection of cytoplasmic yeast virus like elements encoding toxin/antitoxin systems involves a nuclear barrier for immunity gene expression. PLoS Genet. **11**, e1005005. (10.1371/journal.pgen.1005005) 25973601 PMC4431711

[145] Taylor BP, Cortez MH, Weitz JS. 2014 The virus of my virus is my friend: ecological effects of virophage with alternative modes of coinfection. J. Theor. Biol. **354**, 124-136. (10.1016/j.jtbi.2014.03.008) 24662503

[146] Luksa J, Ravoityte B, Konovalovas A, Aitmanaite L, Butenko A, Yurchenko V, Serva S, Servienė E. 2017 Different metabolic pathways are involved in response of *Saccharomyces cerevisiae* to L-A and M viruses. Toxins **9**, 233. (10.3390/toxins9080233) 28757599 PMC5577567

[147] Wilson AJ, Xu J. 2012 Mitochondrial inheritance: diverse patterns and mechanisms with an emphasis on fungi. Mycology **3**, 158-166.

[148] Tipper DJ, Schmitt MJ. 1991 Yeast dsRNA viruses - replication and killer phenotypes. Mol. Microbiol. **5**, 2331-2338. (10.1111/j.1365-2958.1991.tb02078.x) 1665194

[149] Barr CM, Neiman M, Taylor DR. 2005 Inheritance and recombination of mitochondrial genomes in plants, fungi and animals. New Phytol. **168**, 39-50. (10.1111/j.1469-8137.2005.01492.x) 16159319

[150] Bussey H, Vernet T, Sdicu AM. 1988 Mutual antagonism among killer yeasts - competition between K1 and K2 killers and a novel cDNA-based K1-K2 killer strain of *Saccharomyces cerevisiae*. Can. J. Microbiol. **34**, 38-44. (10.1139/m88-007) 3288316

[151] Schmitt MJ, Breinig F. 2002 The viral killer system in yeast: from molecular biology to application. FEMS Microbiol. Rev. **26**, 257-276. (10.1111/j.1574-6976.2002.tb00614.x) 12165427

[152] Quintero-Blanco J, Delodi E, Garzon A, Jimenez J. 2022 Sexually-driven combinatorial diversity in native *Saccharomyces* wine yeasts. Fermentation-Basel **8**, 569. (10.3390/fermentation8100569)

[153] Heath KD. 2010 Intergenomic epistasis and coevolutionary constraint in plants and rhizobia. Evolution **64**, 1446-1458. 20002161 10.1111/j.1558-5646.2009.00913.x

[154] Golubev VI, Churkina LG. 2001 Specificity of sensitivity to mycocin from *Tilletiopsis flava* BKM Y-2838. Mikrobiologiia **70**, 51-54. 11338837

[155] Golubev VI, Nakase T. 1998 Mycocinogeny in *Bullera* genus: killer activity of *Bullera unica* and intragenus killer-sensitive relationships. Mikrobiologiia **67**, 225-230. 9662699

[156] Golubev WI, Pfeiffer I, Golubeva EW. 2006 Mycocin production in *Pseudozyma tsukubaensis*. Mycopathologia **162**, 313-316. (10.1007/s11046-006-0065-2) 17039280

[157] Goto K, Fukuda H, Kichise K, Kitano K, Hara S. 1991 Cloning and nucleotide-sequence of the Khs killer gene of *Saccharomyces cerevisiae*. Agr. Biol. Chem. Tokyo **55**, 1953-1958. 1368726

[158] Goto K, Iwase T, Kichise K, Kitano K, Totuka A, Obata T, Hara S. 1990 Isolation and properties of a chromosome-dependent Khr killer toxin in *Saccharomyces cerevisiae*. Agr. Biol. Chem. Tokyo **54**, 505-509. 19130676

[159] Schneider J, Rupp O, Trost E, Jaenicke S, Passoth V, Goesmann A, Tauch A, Brinkrolf K. 2012 Genome sequence of *Wickerhamomyces anomalus* DSM 6766 reveals genetic basis of biotechnologically important antimicrobial activities. FEMS Yeast Res. **12**, 382-386. (10.1111/j.1567-1364.2012.00791.x) 22292503

[160] Brockhurst MA, Harrison E. 2022 Ecological and evolutionary solutions to the plasmid paradox. Trends Microbiol. **30**, 534-543. (10.1016/j.tim.2021.11.001) 34848115

[161] Itoh T, Martin W, Nei M. 2002 Acceleration of genomic evolution caused by enhanced mutation rate in endocellular symbionts. Proc. Natl Acad. Sci. USA **99**, 12 944-8. (10.1073/pnas.192449699) PMC130565 12235368

[162] Moran NA. 1996 Accelerated evolution and Muller's rachet in endosymbiotic bacteria. Proc. Natl Acad. Sci. USA **93**, 2873-2878. (10.1073/pnas.93.7.2873) 8610134 PMC39726

[163] Woolfit M, Bromham L. 2003 Increased rates of sequence evolution in endosymbiotic bacteria and fungi with small effective population sizes. Mol. Biol. Evol. **20**, 1545-1555. (10.1093/molbev/msg167) 12832648

[164] Dunham M, Gartenberg M, Brown G. 2015 Methods in yeast genetics and genomics. New York, NY: Cold Spring Harbor Laboratory Press.

[165] Colegrave N. 2002 Sex releases the speed limit on evolution. Nature **420**, 664-666. (10.1038/nature01191) 12478292

[166] Roze D. 2012 Disentangling the benefits of sex. PLoS Biol. **10**, e1001321. (10.1371/journal.pbio.1001321) 22563302 PMC3341332

[167] Butlin R. 2002 Evolution of sex: the costs and benefits of sex: new insights from old asexual lineages. Nat. Rev. Genet. **3**, 311-317. (10.1038/nrg749) 11967555

[168] Otto SP, Lenormand T. 2002 Resolving the paradox of sex and recombination. Nat. Rev. Genet. **3**, 252-261. (10.1038/nrg761) 11967550

[169] Charlesworth B, Barton NH. 1996 Recombination load associated with selection for increased recombination. Genet. Res. **67**, 27-41. (10.1017/S0016672300033450) 8919888

[170] Barton NH, Charlesworth B. 1998 Why sex and recombination? Science **281**, 1986-1990. (10.1126/science.281.5385.1986) 9748151

[171] Vogan AA, Ament-Velasquez SL, Bastiaans E, Wallerman O, Saupe SJ, Suh A, Johannesson H. 2021 The Enterprise, a massive transposon carrying Spok meiotic drive genes. Genome Res. **31**, 789-798. (10.1101/gr.267609.120) 33875482 PMC8092012

[172] Heitman J, Sun S, James TY. 2013 Evolution of fungal sexual reproduction. Mycologia **105**, 1-27. (10.3852/12-253) 23099518

[173] Ni M, Feretzaki M, Sun S, Wang XY, Heitman J. 2011 Sex in fungi. Annu. Rev. Genet. **45**, 405-430. (10.1146/annurev-genet-110410-132536) 21942368 PMC3310392

[174] Neiman AM. 2011 Sporulation in the budding yeast *Saccharomyces cerevisiae*. Genetics **189**, 737-765. (10.1534/genetics.111.127126) 22084423 PMC3213374

[175] Nieuwenhuis BPS, James TY. 2016 The frequency of sex in fungi. Phil. Trans. R. Soc. B **371**, 20150540. (10.1098/rstb.2015.0540) 27619703 PMC5031624

[176] Pringle A, Taylor JW. 2002 The fitness of filamentous fungi. Trends Microbiol. **10**, 474-481. (10.1016/S0966-842X(02)02447-2) 12377558

[177] Rowley PA. 2017 The frenemies within: viruses, retrotransposons and plasmids that naturally infect *Saccharomyces* yeasts. Yeast **34**, 279-292. (10.1002/yea.3234) 28387035

[178] Bruce JB, West SA, Griffin AS. 2017 Bacteriocins and the assembly of natural *Pseudomonas fluorescens* populations. J. Evol. Biol. **30**, 352-360. (10.1111/jeb.13010) 28000957 PMC6849615

[179] Travers-Cook TJ, Jokela J, Buser CC. 2023 The evolutionary ecology of fungal killer phenotypes. Figshare. (10.6084/m9.figshare.c.6761810) PMC10427833 37583325

